# Reusable and Antibacterial Polymer‐Based Nanocomposites for the Adsorption of Dyes and the Visible‐Light‐Driven Photocatalytic Degradation of Antibiotics

**DOI:** 10.1002/gch2.202200076

**Published:** 2022-08-25

**Authors:** Jiao Wang, Massimo Sgarzi, Zuzana Němečková, Jiří Henych, Nadia Licciardello, Gianaurelio Cuniberti

**Affiliations:** ^1^ Institute for Materials Science Max Bergmann Center of Biomaterials and Dresden Center for Nanoanalysis TU Dresden 01062 Dresden Germany; ^2^ Institute of Inorganic Chemistry Czech Academy of Sciences Husinec‐Řež 1001 Řež 250 68 Czech Republic; ^3^ Faculty of Environment Jan Evangelista Purkyně University Pasteurova 3632/15 Ústí nad Labem 400 96 Czech Republic; ^4^ Present address: Department of Molecular Sciences and Nanosystems Ca’ Foscari University of Venice Via Torino 155 30172 Venezia Mestre Italy; ^5^ Present address: Department of Drug and Health Sciences University of Catania Viale Andrea Doria 6 95125 Catania Italy

**Keywords:** adsorption, dyes and antibiotics, multifunctionality, recycling, visible‐light‐driven photocatalysis

## Abstract

Adsorption and advanced oxidation processes, especially photocatalysis, are amongst the most common water treatment methodologies. Unfortunately, using each of these techniques independently does not fully eliminate the pollutants of diverse nature, which are present in wastewater. Here, an avenue for multifunctional materials for water treatment is opened by reporting for the first time the preparation, characterization, and study of the properties of a novel multifunctional nanocomposite with both adsorption and visible‐light‐driven photocatalysis abilities. These multifunctional nanocomposites, namely iron (II, III) oxide/poly(*N*‐isopropylacrylamide‐*co*‐methacrylic acid)/silver‐titanium dioxide (Fe_3_O_4_/P(NIPAM‐*co*‐MAA)/Ag‐TiO_2_), are prepared by combining magnetic polymeric microspheres (Fe_3_O_4_/P(NIPAM‐*co*‐MAA)) with silver‐decorated titanium dioxide nanoparticles (Ag‐TiO_2_ NPs). Cationic dyes, such as basic fuchsin (BF), can be adsorbed by the nanocomposites thanks to the carboxylic groups of Fe_3_O_4_/P(NIPAM‐*co*‐MAA) microspheres. Concomitantly, the presence of Ag‐TiO_2_ NPs endows the system with the visible‐light‐driven photocatalytic degradation ability toward antibiotics such as ciprofloxacin (CIP) and norfloxacin (NFX). Furthermore, the proposed nanocomposites show antibacterial activity toward *Escherichia coli* (*E. coli*), thanks to the presence of silver nanoparticles (Ag NPs). Due to the superparamagnetic properties of iron (II, III) oxide nanoparticles (Fe_3_O_4_ NPs), the nanocomposites can be also recycled and reused, after the cleaning process, by using an external magnetic field.

## Graphical abstract

Multifunctional iron (II, III) oxide/poly(*N*‐isopropylacrylamide‐*co*‐methacrylic acid)/silver‐titanium dioxide (Fe_3_O_4_/P(NIPAM‐*co*‐MAA)/Ag‐TiO_2_) nanocomposites exhibit outstanding adsorption capability toward the dye basic fuchsin (BF) and good visible‐light‐driven photocatalytic activity toward the antibiotics ciprofloxacin (CIP) and norfloxacin (NFX). The nanocomposites, which can be regenerated and reused, show antibacterial activity toward *Escherichia coli* demonstrating their promising utilization as recyclable adsorbents, photocatalysts, and antibacterial materials for wastewater treatment.

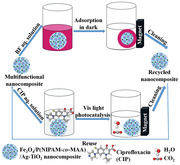

## 1 Introduction

Water pollution refers to the physical, chemical, or biological contamination of water bodies that affects organisms,^[^ ^1^ ^]^ and is a major environmental issue posing severe threats to human and wildlife health.^[^ ^2^, ^3^ ^]^ Among all kinds of pollutants, especially dyes, antibiotics, and bacteria are remarkably harmful.^[^ ^4^ ^]^ Dyes and antibiotics are anthropogenic chemical pollutants contained in industrial, municipal, and hospital wastewater:^[^ ^5^, ^6^, ^7^, ^8^ ^]^ while most of the dyes are genotoxic, cytotoxic, or carcinogenic,^[^ ^9^, ^10^, ^11^ ^]^ antibiotics are conducive to antibiotic‐resistant bacteria.^[^ ^12^ ^]^ On the other hand, bacteria are common pollutants in wastewater treatment plants^[^ ^13^, ^14^, ^15^ ^]^ and can cause several diseases, including diarrhea and gastrointestinal infections.^[^ ^13^, ^16^ ^]^ Among the tertiary treatment techniques, adsorption^[^ ^9^, ^17^ ^]^ and advanced oxidation processes (AOPs)^[^ ^18^ ^]^—especially photocatalysis—are particularly interesting. Adsorption has several advantages over other techniques, such as lower cost and easier operation,^[^ ^19^ ^]^ and has been extensively used to remove organic or inorganic pollutants in wastewater.^[^ ^20^ ^]^ Photocatalysis, one of the most common AOPs, makes use of semiconductor materials (photocatalysts) which can be photoactivated, depending on the energy of their bandgap, by ultraviolet, visible, or infrared light to induce the oxidative degradation of the pollutants.^[^ ^21^ ^]^ The advantages of photocatalytic materials for pollutants remediation are the ability to remove persistent organic pollutants and, ideally, the avoidance of secondary pollutants formation.^[^ ^22^ ^]^

So far, many scientific reports deal with the removal of one single pollutant with one material and one technique.^[^ ^23^, ^24^ ^]^ However, real wastewater samples are extremely complex, comprising different kinds of pollutants,^[^ ^1^, ^2^ ^]^ which require different techniques to be removed in an efficient and environmentally friendly way. In this context, multifunctional composite materials such as Ag‐TiO_2_/poly(vinylidene fluoride‐*co*‐hexafluoropropylene) (Ag‐TiO_2_/PVDF‐HFP) membranes^[^ ^25^ ^]^ and 9,9′‐bifluorenylidene‐based conjugated microporous/mesoporous polymers (BF‐CMPs)^[^ ^26^ ^]^ were reported. The reported Ag‐TiO_2_/PVDF‐HFP membrane exhibited photocatalytic activity toward norfloxacin (NFX) and antibacterial activity toward *Escherichia coli* (*E. coli*) and *Staphylococcus epidermidis*.^[^ ^25^ ^]^ However, the ability of these composites to eliminate organic dyes from wastewater was not studied. Although BF‐CMPs exhibited high adsorption capacity and photocatalytic degradation toward Rhodamine B,^[^ ^26^ ^]^ their adsorption capacity toward other pollutants was not fully reported.

Here, Fe_3_O_4_/P(NIPAM‐*co*‐MAA)/Ag‐TiO_2_ nanocomposites were prepared in order to obtain multifunctional materials able to perform more than one task. The nanocomposites were obtained by combining Fe_3_O_4_/P(NIPAM‐*co*‐MAA) microspheres^[^ ^27^ ^]^ and Ag‐TiO_2_ NPs.^[^ ^28^ ^]^ Fe_3_O_4_/P(NIPAM‐*co*‐MAA) microspheres were endowed with the ability to adsorb cationic organic dyes, such as basic fuchsin (BF), and possessed magnetic properties, which have been reported in a previous work.^[^ ^27^ ^]^ In addition, in our previous publication,^[^ ^28^ ^]^ Ag‐TiO_2_ NPs with enhanced visible‐light‐driven photocatalytic degradation of antibiotics and antibacterial activity were prepared. Hence, the nanocomposites prepared in the present work comprise all the properties of their single components, namely dyes adsorption, visible‐light‐driven photocatalytic activity toward antibiotics, and antibacterial properties. The cationic dye BF could be adsorbed by the nanocomposites thanks to the functional carboxylic groups present on the Fe_3_O_4_/P(NIPAM‐*co*‐MAA) microspheres. Due to the presence of Ag‐TiO_2_ NPs on the one hand, the nanocomposites showed the ability to degrade the antibiotics ciprofloxacin (CIP) and NFX under visible‐light irradiation, and, on the other hand, presented good antibacterial activity toward *E. coli*. Furthermore, thanks to the superparamagnetic properties of Fe_3_O_4_ NPs, the nanocomposites could be easily collected and recycled from the treated water by using an external magnetic field.

## 2 Results and Discussion

### 2.1 Preparation and Characterization of Fe_3_O_4_/P(NIPAM‐*co*‐MAA)/Ag‐TiO_2_ Nanocomposites

The preparation pathway for the proposed Fe_3_O_4_/P(NIPAM‐*co*‐MAA)/Ag‐TiO_2_ nanocomposites is schematically illustrated in Scheme S1, Supporting Information, and involves the combination of magnetic Fe_3_O_4_/P(NIPAM‐*co*‐MAA) microspheres, previously reported from Wang et al.,^[^ ^27^ ^]^ and Ag‐TiO_2_ NPs, as previously reported from Wang et al.^[^ ^28^ ^]^ The details are reported in the Experimental Section. The possible explanation about the interaction between the Fe_3_O_4_/P(NIPAM‐*co*‐MAA) microspheres and the Ag‐TiO_2_ NPs in the nanocomposites is that the carboxylate groups on Fe_3_O_4_/P(NIPAM‐*co*‐MAA) microspheres may transfer their acidic hydrogen to the surface of Ag‐TiO_2_ NPs, forming a bridging hydroxyl group and a chemisorbed methacrylate moiety.^[^ ^29^, ^30^ ^]^

**Figure 1:**
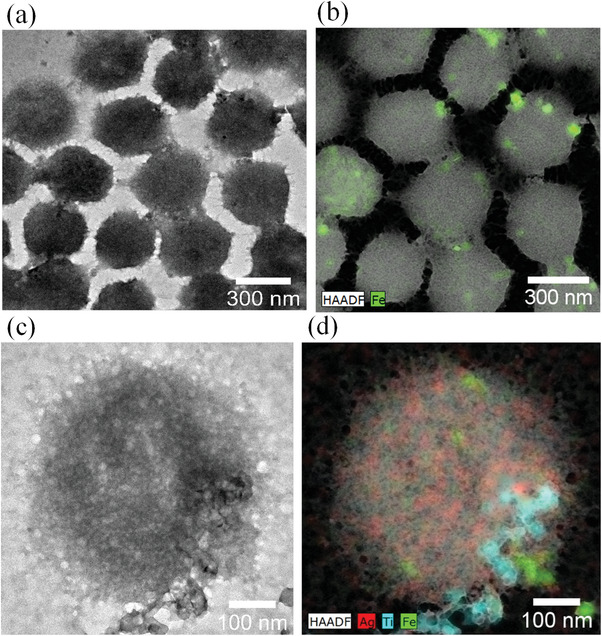
a) HR‐TEM image of Fe_3_O_4_/P(NIPAM‐*co*‐MAA) microspheres. b) EDS map of Fe_3_O_4_/P(NIPAM‐*co*‐MAA) microspheres with Fe (green) atomic distribution. c) HR‐TEM image of Fe_3_O_4_/P(NIPAM‐*co*‐MAA)/Ag‐TiO_2_ nanocomposites. d) EDS map of Fe_3_O_4_/P(NIPAM‐*co*‐MAA)/Ag‐TiO_2_ nanocomposites with Fe (green), Ti (blue), and Ag (red) atomic distribution.

**Figures** **1**a,c show the high‐resolution transmission electron microscopy (HR‐TEM) images of Fe_3_O_4_/P(NIPAM‐*co*‐MAA) microspheres and Fe_3_O_4_/P(NIPAM‐*co*‐MAA)/Ag‐TiO_2_ nanocomposites, respectively. The images show that, after the combination of the Fe_3_O_4_/P(NIPAM‐*co*‐MAA) microspheres with Ag‐TiO_2_ NPs, the microspheres’ structure is maintained. Furthermore, the average size of Fe_3_O_4_/P(NIPAM‐*co*‐MAA)/Ag‐TiO_2_ nanocomposites is 398 ± 9 nm, which is comparable with the average size of Fe_3_O_4_/P(NIPAM‐*co*‐MAA) microspheres (410 ± 11 nm). The energy dispersive X‐ray spectroscopy (EDS) map of Fe_3_O_4_/P(NIPAM‐*co*‐MAA) microspheres with iron (Fe) atomic distribution is shown in Figure 1b indicating the presence of Fe_3_O_4_ NPs in the microspheres. Figure 1d shows the EDS map of Fe_3_O_4_/P(NIPAM‐*co*‐MAA)/Ag‐TiO_2_ nanocomposites with Fe, titanium (Ti), and silver (Ag) atomic distribution, which confirms the presence of the three elements in the nanocomposites. A more detailed and average quantification of the three elements in the Fe_3_O_4_/P(NIPAM‐*co*‐MAA)/Ag‐TiO_2_ nanocomposites is reported in Figure S1 and Table S1, Supporting Information.

In order to evaluate the chemical composition of the nanocomposites, attenuated total reflectance Fourier‐transform infrared (ATR‐FTIR) spectra of Ag‐TiO_2_ NPs, Fe_3_O_4_/P(NIPAM‐*co*‐MAA) microspheres, and Fe_3_O_4_/P(NIPAM‐*co*‐MAA)/Ag‐TiO_2_ nanocomposites were acquired (Figure S2, Supporting Information). As reported previously,^[^ ^27^ ^]^ the IR spectrum of Fe_3_O_4_/P(NIPAM‐*co*‐MAA) microspheres in Figure S2 (blue line and short description), Supporting Information, shows that all necessary components have been incorporated into the internal structure of the Fe_3_O_4_/P(NIPAM‐*co*‐MAA) microspheres. Fe_3_O_4_/P(NIPAM‐*co*‐MAA)/Ag‐TiO_2_ nanocomposites exhibit an IR spectrum (Figure S2, green line, Supporting Information) similar to that of Fe_3_O_4_/P(NIPAM‐*co*‐MAA) microspheres, suggesting that the chemical structure of Fe_3_O_4_/P(NIPAM‐*co*‐MAA) microspheres did not significantly change after combination with Ag‐TiO_2_ NPs. The successful preparation of Ag‐TiO_2_ NPs has been demonstrated in our previous publication.^[^ ^28^ ^]^ However, it is difficult to evaluate the presence of Ag‐TiO_2_ NPs in the nanocomposites by the exclusive use of ATR‐FTIR measurements. Ultraviolet–visible (UV–vis) absorption spectra of Ag‐TiO_2_ NPs, Fe_3_O_4_/P(NIPAM‐*co*‐MAA) microspheres, and Fe_3_O_4_/P(NIPAM‐*co*‐MAA)/Ag‐TiO_2_ nanocomposites were also recorded and compared (Figure S3, Supporting Information) to further confirm the presence of Ag‐TiO_2_ NPs in the nanocomposites and to investigate the optical properties of the samples. Ag‐TiO_2_ NPs present a TiO_2_ absorption edge at 380 nm^[^ ^31^, ^32^ ^]^ and a weak and broad absorption band between 400 and 650 nm due to the localized surface plasmon resonance of silver nanoparticles (Ag NPs).^[^ ^32^ ^]^ These characteristics are present also in the spectrum of the Fe_3_O_4_/P(NIPAM‐*co*‐MAA)/Ag‐TiO_2_ nanocomposites, while the Fe_3_O_4_/P(NIPAM‐*co*‐MAA) microspheres exhibit no distinct absorption band in the 200–800 nm range.

**Figure 2:**
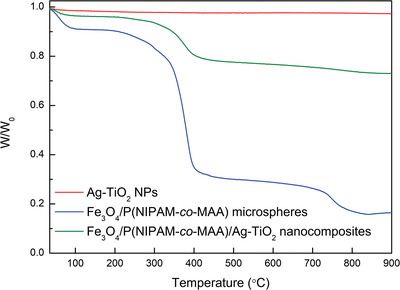
TGA measurements for Ag‐TiO_2_ NPs (red line), Fe_3_O_4_/P(NIPAM‐*co*‐MAA) microspheres (blue line), and Fe_3_O_4_/P(NIPAM‐*co*‐MAA)/Ag‐TiO_2_ nanocomposites (green line).

Thermogravimetric analyses (TGA) of Ag‐TiO_2_ NPs, Fe_3_O_4_/P(NIPAM‐*co*‐MAA) microspheres, and Fe_3_O_4_/P(NIPAM‐*co*‐MAA)/Ag‐TiO_2_ nanocomposites are shown in **Figure** **2**, and the corresponding weight losses are reported in **Table** **1**. The results show that, after heating up from 30 to 900 °C, only a 2% weight loss occurred for Ag‐TiO_2_ NPs, as previously reported for this kind of samples.^[^ ^33^ ^]^ There were, instead, three weight losses for both Fe_3_O_4_/P(NIPAM‐*co*‐MAA) microspheres and Fe_3_O_4_/P(NIPAM‐*co*‐MAA)/Ag‐TiO_2_ nanocomposites in the range from 30 to 900 °C. The first weight loss for Fe_3_O_4_/P(NIPAM‐*co*‐MAA) microspheres (10% at 60 °C, Figure S4a, Supporting Information) and Fe_3_O_4_/P(NIPAM‐*co*‐MAA)/Ag‐TiO_2_ nanocomposites (3% at 55 °C, Figure S4b, Supporting Information) appeared in the range from 30 to 100 °C, which is ascribable to the loss of water molecules.^[^ ^34^ ^]^ The second weight loss occurred in the range of 200–400 °C, due to the thermal degradation of the polymer skeleton.^[^ ^35^ ^]^ This weight loss was 58% at 380 °C for the microspheres (Figure S4a, Supporting Information, and Table 1) and 18% at 373 °C for the nanocomposites (Figure S4b, Supporting Information, and Table 1), showing that the nanocomposites are thermally more stable than the microspheres. This phenomenon might be ascribable to the lower amount of polymer in the nanocomposites compared to the amount of polymer in the microspheres (when comparing the same amount of the two samples) or to a higher stabilization due to the presence of Ag‐TiO_2_ NPs.^[^ ^36^ ^]^ The third weight loss occurred in the range of 700–850 °C. This weight loss was 16% at 750 °C for the microspheres (Figure S4a, Supporting Information, and Table 1) and 6% at 805 °C for the nanocomposites (Figure S4b, Supporting Information, and Table 1). The percentage residual weight (*R*%) at 850 °C was 16% for the microspheres, which is ascribable to the residual Fe_3_O_4_ NPs. The higher value of *R*% (73%) registered for the nanocomposites confirmed the presence of both residual Ag‐TiO_2_ NPs and Fe_3_O_4_ NPs in the nanocomposites.^[^ ^36^ ^]^

**Table 1:** TGA data of Ag‐TiO_2_ NPs, Fe_3_O_4_/P(NIPAM‐*co*‐MAA) microspheres, and Fe_3_O_4_/P(NIPAM‐*co*‐MAA)/Ag‐TiO_2_ nanocomposites

Samples	Temperature range [°C]	Weight loss [wt%]	Percentage residual weight *R*% at 850 °C
Ag‐TiO_2_ NPs	30–100 200–420 700–900	2 0 0	98
Fe_3_O_4_/P(NIPAM‐*co*‐MAA) microspheres	30–100 200–420 700–900	10 58 16	16
Fe_3_O_4_/P(NIPAM‐*co*‐MAA)/Ag‐TiO_2_ nanocomposites	30–100 200–420 700–900	3 18 6	73

**Figure 3:**
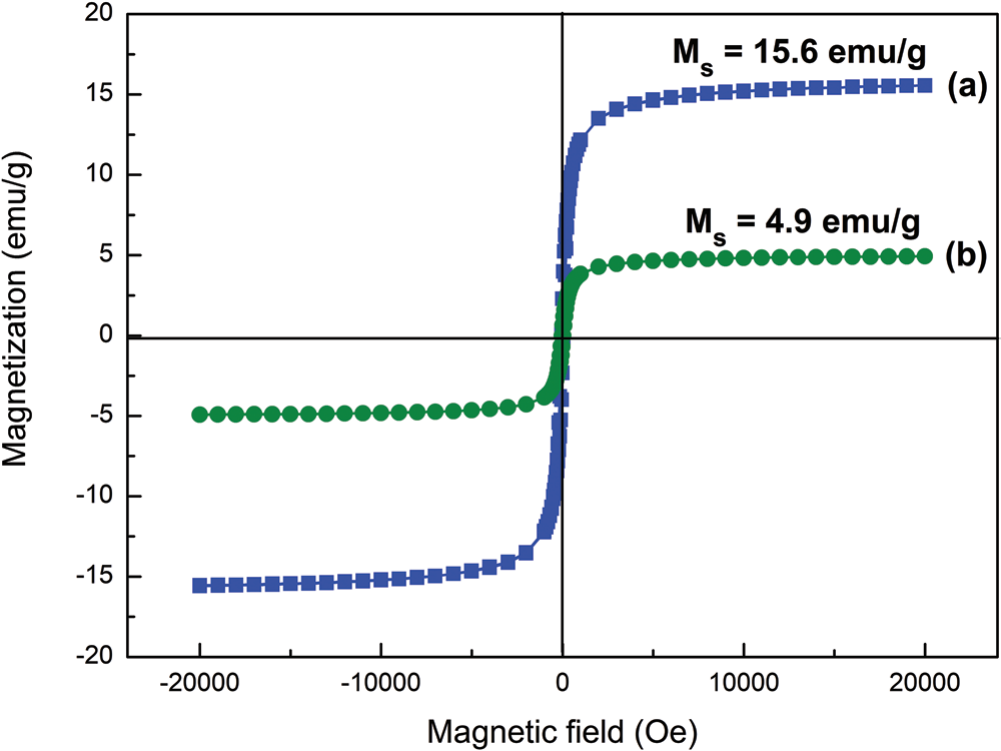
Magnetization curves of a) Fe_3_O_4_/P(NIPAM‐*co*‐MAA) microspheres (blue line) and b) Fe_3_O_4_/P(NIPAM‐*co*‐MAA)/Ag‐TiO_2_ nanocomposites (green line).

The magnetic properties of Fe_3_O_4_/P(NIPAM‐*co*‐MAA) microspheres and Fe_3_O_4_/P(NIPAM‐*co*‐MAA)/Ag‐TiO_2_ nanocomposites were characterized by vibrating sample magnetometer (VSM) analyses (**Figure** **3**). No hysteresis was observed in both microspheres and nanocomposites samples, and the remanent magnetization and the coercivity were equal to zero. These results are in line with the superparamagnetic properties of Fe_3_O_4_ NPs.^[^ ^27^ ^]^ The saturation magnetization (*M*_s_) of the microspheres and the nanocomposites was 15.6 and 4.9 emu g^−1^ at 5000 Oe, respectively, where the corresponding magnetic susceptibility (χ) was 3.12 × 10^−3^ and 9.8 × 10^–4^ emu g^−1^ Oe^−1^.^[^ ^37^ ^]^ The ratio between the *M*_s_ values for the microspheres and the nanocomposites was ≈3:1, corresponding to the ratio between the Fe_3_O_4_ NPs mass in the microspheres and the Fe_3_O_4_ NPs mass in the nanocomposites. Indeed, the amount used for the VSM measurements was identical for the microspheres and the nanocomposites, but the mass ratio of Fe_3_O_4_ in the microspheres and in the nanocomposites was 3:1. Hence, the magnetic properties of Fe_3_O_4_ NPs were not affected by the addition of Ag‐TiO_2_ NPs. Following these results, it was possible to demonstrate that Fe_3_O_4_/P(NIPAM‐*co*‐MAA)/Ag‐TiO_2_ nanocomposites possess superparamagnetic properties.

### 2.2 Adsorption Capability of Fe_3_O_4_/P(NIPAM‐*co*‐MAA)/Ag‐TiO_2_ Nanocomposites

**Figure 4:**
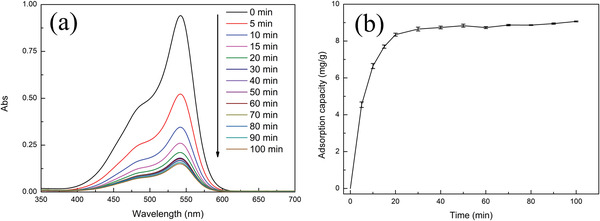
a) Representative temporal variations of the BF absorption spectrum in water and b) adsorption kinetic curve of BF solution in water during the adsorption on Fe_3_O_4_/P(NIPAM‐*co*‐MAA)/Ag‐TiO_2_ nanocomposites under dark conditions (initial concentration of BF: 5 mg L^−1^, concentration of the nanocomposites: 460 mg L^−1^, number of replicates *n* = 3).

Once the nanocomposites were characterized, their multifunctionality was tested. First, the adsorption ability of the nanocomposites was assessed. BF (chemical structure in Figure S5a, Supporting Information) is one of the common organic dyes found in wastewater and was used as a model compound in this work to evaluate the adsorption capability of Fe_3_O_4_/P(NIPAM‐*co*‐MAA)/Ag‐TiO_2_ nanocomposites. **Figure** **4**a shows the temporal variation of the BF absorption spectrum during the adsorption experiment on the nanocomposites. In Figure 4b, the adsorption kinetics curve of BF is reported and clearly indicates that BF reached an adsorption–desorption equilibrium on the nanocomposites within 30 min. The kinetic constants obtained from the pseudo‐first‐order (Equation (2) and Experimental Section) and pseudo‐second‐order (Equation (3) and Experimental Section) models are listed in **Table** **2**, while the corresponding fitting are reported in Figure S6, Supporting Information. According to the pseudo‐first‐order model, the adsorption rate is directly proportional to the difference between the saturation concentration and the amount of adsorbate uptaken with time.^[^ ^38^, ^39^ ^]^ According to the pseudo‐second‐order model, the adsorption rate depends on the adsorption capacity and not on the concentration of adsorbate.^[^ ^38^, ^39^ ^]^ Figure S6, Supporting Information, and Table 2 indicate that the kinetic data of BF by the nanocomposites are better fitted with the pseudo‐second‐order model than with the pseudo‐first‐order. There are two pieces of evidence to support the above conclusion: the correlation coefficient value of the pseudo‐second‐order model (*R* ^2^ = 0.9982) is higher than the one of the pseudo‐first‐order model (*R* ^2^ = 0.8020), and the theoretical equilibrium adsorption capacity of the pseudo‐second‐order (9.19 mg g^−1^) is closer to the actual equilibrium adsorption capacity (*q*_e,exp_ = 9.07 mg g^−1^). Therefore, the adsorption kinetics of the nanocomposites toward BF is more in line with the pseudo‐second‐order model, indicating that the rate‐determining step in the adsorption process is the interaction of BF with the adsorption sites of the nanocomposites.^[^ ^38^, ^39^, ^40^, ^41^ ^]^

**Table 2:** Model parameters of adsorption kinetics for BF adsorption by Fe_3_O_4_/P(NIPAM‐*co*‐MAA)/Ag‐TiO_2_ nanocomposites

Dye	Pseudo‐first‐order model				Pseudo‐second‐order model		
	*q*_e,exp_[mg g^−1^]	*q*_e_[mg g^−1^]	*k*_1_ [min^−1^]	*R* ^2^	*q*_e_[mg g^−1^]	*k*_2_[g mg^−1^ min^−1^]	*R* ^2^
BF	9.07	3.15	4.09 × 10^−2^	0.8020	9.19	4.22 × 10^−2^	0.9982

**Figure 5:**
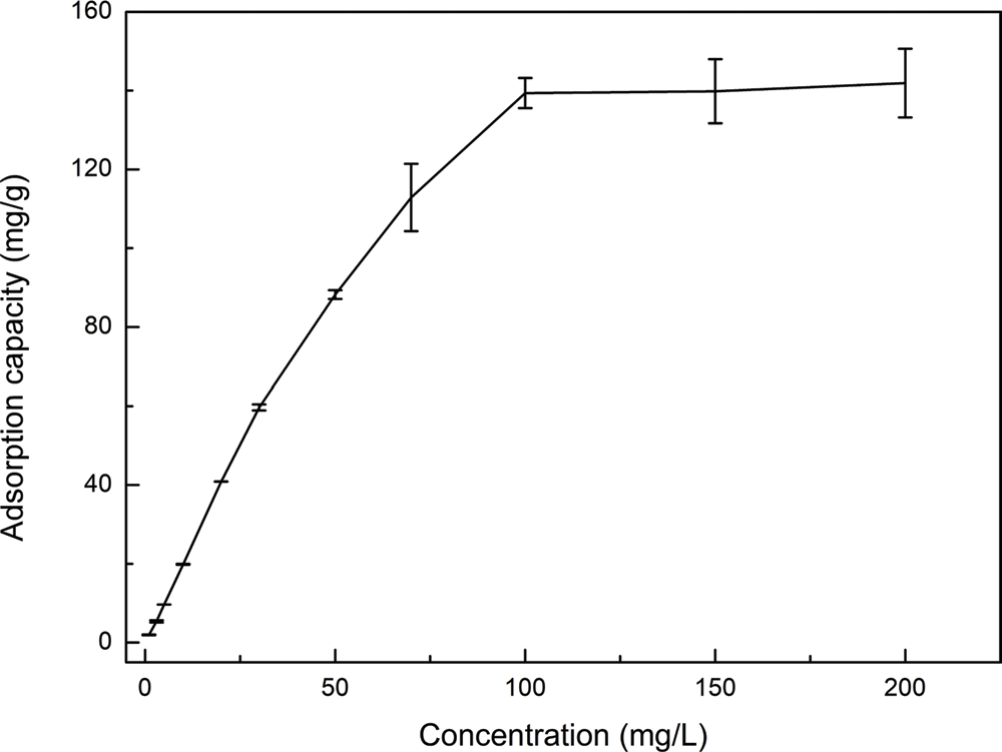
Adsorption isotherm curve of BF by Fe_3_O_4_/P(NIPAM‐*co*‐MAA)/Ag‐TiO_2_ nanocomposites under dark conditions (concentration of the nanocomposites: 460 mg L^−1^, number of replicates *n* = 2).

The experimental adsorption isotherm for BF adsorption on the nanocomposites (**Figure** **5**) indicates that the maximum adsorption capacity of the nanocomposites toward BF reached 150 mg g^−1^ at room temperature after 100 min of adsorption. Langmuir and Freundlich adsorption isotherms obtained using Equations (4) and (5) are presented in Figure S7, Supporting Information, and the corresponding obtained parameters are in **Table** **3**. In the Langmuir adsorption model, the adsorption of the adsorbate is limited to just one monolayer, and the model implies that the adsorption of the adsorbate molecules takes place on the surface of the adsorbent with the same adsorption energies.^[^ ^42^ ^]^ More in detail, it states that the adsorbent sites are all homogeneous, which means that there are no interactions between adsorbates, and each adsorbate occupies a single site. The Freundlich adsorption model implies, instead, a multilayer adsorption, which takes place with different adsorption energies,^[^ ^42^ ^]^ because the sites on the surface of the adsorbent are heterogeneous. Figure S7, Supporting Information, and Table 3 indicate that the data are better fitted with the Langmuir adsorption model than with the Freundlich one. This is an additional proof that the sites on the surface of the adsorbent are homogeneous and it is in full agreement with the previous finding that the rate‐determining step in the adsorption process is the interaction of BF with the adsorption sites of the nanocomposites. Therefore, it can be presumed that the adsorption of the positively charged BF on the nanocomposites occurs through the electrostatic attraction of BF to the negatively charged carboxylate groups on the nanocomposites.

**Table 3:** Model parameters of the Langmuir and Freundlich models for BF adsorption on Fe_3_O_4_/P(NIPAM‐*co*‐MAA)/Ag‐TiO_2_ nanocomposites

Dye	Langmuir constants			Freundlich constants		
	*K*_L_ [L mg^−1^]	*q*_m_ [mg g^−1^]	*R* ^2^	*K*_F_ [mg^1−1/n^ L^1/n^ g^−1^]	1/*n*	*R* ^2^
BF	0.1405	150.38	0.9942	15.12	0.5928	0.8444

Subsequently, the adsorption capability of Fe_3_O_4_/P(NIPAM‐*co*‐MAA)/Ag‐TiO_2_ nanocomposites toward CIP (chemical structure in Figure S5b, Supporting Information) and NFX (chemical structure in Figure S5c, Supporting Information) was assessed under dark conditions. 50 mL of CIP or NFX solution (5 mg L^−1^) were put into a beaker with 23 mg of the nanocomposites under stirring and dark conditions. After 180 min, a negligible adsorption of CIP (2%, Figure S8, Supporting Information) and NFX (1%, Figure S9, Supporting Information) was observed. Thus, it was possible to conclude that the nanocomposites show non‐significant adsorption capability toward CIP and NFX, and another method (such as photocatalysis) might be required to remove these pollutants from water employing these nanocomposites. This behavior might be ascribed to the different chemical structure of the antibiotics when compared to the BF dye.

### 2.3 Visible‐Light‐Driven Photocatalytic Activity of Fe_3_O_4_/P(NIPAM‐*co*‐MAA)/Ag‐TiO_2_ Nanocomposites

Once the adsorption ability of the Fe_3_O_4_/P(NIPAM‐*co*‐MAA)/Ag‐TiO_2_ nanocomposites was tested, their capability to degrade antibiotics by photocatalysis was investigated in order to prove their multifunctionality. The common antibiotics CIP and NFX were chosen as models in this study. As we reported in our former publication,^[^ ^28^ ^]^ Ag‐TiO_2_ NPs can degrade CIP and NFX under visible‐light irradiation. Therefore, we expected that CIP and NFX could be degraded also by the nanocomposites under visible‐light irradiation, thanks to the presence of Ag‐TiO_2_ NPs in the nanocomposites. The photocatalytic experiments for the degradation of CIP and NFX by the Fe_3_O_4_/P(NIPAM‐*co*‐MAA)/Ag‐TiO_2_ nanocomposites were performed as follows. The nanocomposites and the antibiotic were mixed for 30 min under stirring and in dark conditions to reach the adsorption–desorption equilibrium. Indeed, as explained previously (Figures S8 and S9, Supporting Information), the antibiotic concentration did not significantly vary after 30 min, which indicates that an adsorption–desorption equilibrium was already reached by this time. Subsequently, the photocatalytic degradation of the antibiotic by the nanocomposites was performed under visible‐light irradiation.

**Figure 6:**
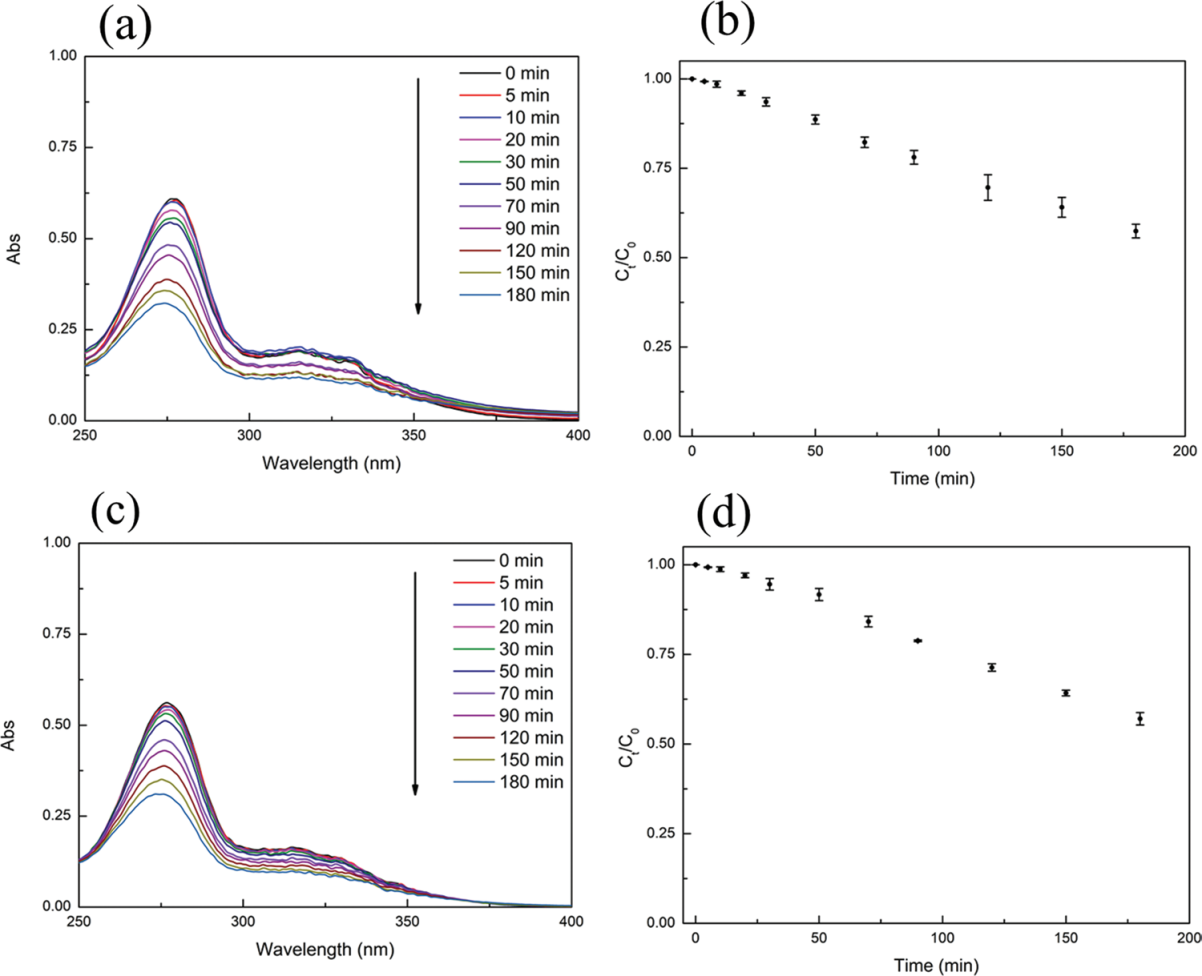
a) Representative temporal variations of the absorption spectrum of CIP under visible‐light irradiation in the presence of Fe_3_O_4_/P(NIPAM‐*co*‐MAA)/Ag‐TiO_2_ nanocomposites. b) Time‐dependent concentration variation of CIP solution upon exposure to visible light in the presence of Fe_3_O_4_/P(NIPAM‐*co*‐MAA)/Ag‐TiO_2_ nanocomposites. c) Representative temporal variations of the absorption spectrum of NFX under visible‐light irradiation in the presence of Fe_3_O_4_/P(NIPAM‐*co*‐MAA)/Ag‐TiO_2_ nanocomposites. d) Time‐dependent variation of the concentration of NFX solution upon exposure to visible light in the presence of Fe_3_O_4_/P(NIPAM‐*co*‐MAA)/Ag‐TiO_2_ nanocomposites (initial concentration of CIP or NFX: 5 mg L^−1^, concentration of the nanocomposites: 460 mg L^−1^). For (b) and (d), the number of replicates is *n* = 3.

**Figure** **6**a,b shows that a significant degradation of CIP occurred in the presence of the nanocomposites under visible‐light irradiation. The degradation efficiency of CIP by the nanocomposites was 47% after 180 min visible‐light irradiation. The application of the pseudo‐first‐order kinetic model (Equation (6)) to the photocatalytic degradation kinetics of CIP by the nanocomposites allowed to calculate the degradation rate constant (*k* = 3.12 × 10^−3^ min^−1^; Figure S10a, Supporting Information, and **Table** **4**). Similar results were obtained for NFX (Figure 6c,d). The degradation efficiency of NFX by the nanocomposites was 47% after 180 min visible‐light irradiation, and the pseudo‐first‐order degradation rate constant was *k* = 3.12 × 10^−3^ min^−1^ (Figure S10b, Supporting Information, and Table 4).

**Table 4:** Photocatalytic degradation efficiency and pseudo‐first‐order degradation rate constants for Fe_3_O_4_/P(NIPAM‐*co*‐MAA)/Ag‐TiO_2_ nanocomposites

Antibiotics	Degradation efficiency	*k* [10^−3^ min^−1^]
CIP NFX	47% 47%	3.12 3.12

Subsequently, in order to determine the photocatalytic degradation activity of Fe_3_O_4_/P(NIPAM‐*co*‐MAA)/Ag‐TiO_2_ nanocomposites toward BF, the nanocomposites were irradiated with visible‐light in the presence of BF. Due to the multifunctional nature of the nanocomposites, it is difficult to assess the contribution of the photocatalytic degradation of BF to the full BF removal by the nanocomposites. Therefore, since the photocatalytic active components of these nanocomposites are the Ag‐TiO_2_ NPs, they were used to perform the experiments of photocatalytic degradation of BF under visible‐light irradiation. These photocatalytic experiments are similar to those for the photocatalytic degradation of antibiotics by the nanocomposites. It is worth noting that the concentration of Ag‐TiO_2_ NPs used in these photocatalytic experiments was 300 mg L^−1^, whose value corresponds to the concentration of Ag‐TiO_2_ NPs contained in 460 mg L^−1^ nanocomposites. Figure S11, Supporting Information, shows the negligible degradation (3%) of BF by the Ag‐TiO_2_ NPs under 180 min visible‐light irradiation. It can be, therefore, concluded that the Ag‐TiO_2_ NPs and, therefore, the nanocomposites cannot degrade BF under visible‐light irradiation. The possible explanation is that BF has a stable triarylmethane structure which is very different from the chemical structure of CIP and NFX, which can conversely be degraded by the nanocomposites.

### 2.4 Reusability of Fe_3_O_4_/P(NIPAM‐*co*‐MAA)/Ag‐TiO_2_ Nanocomposites

**Figure 7:**
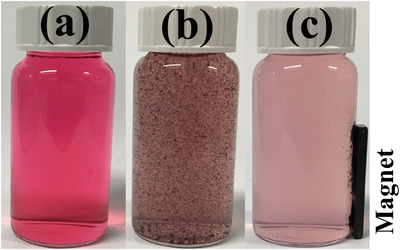
Photographs of the recovering process for Fe_3_O_4_/P(NIPAM‐*co*‐MAA)/Ag‐TiO_2_ nanocomposites through a simple magnetic separation procedure. a) BF solution; b) BF solution in the presence of the nanocomposites; c) Application of an external magnetic field to recover the nanocomposites (initial concentration of BF: 5 mg L^−1^, concentration of the nanocomposites: 460 mg L^−1^).

Similarly to the behavior already reported for the polymer porous microspheres,^[^ ^27^ ^]^ due to the superparamagnetic properties of Fe_3_O_4_ NPs, Fe_3_O_4_/P(NIPAM‐*co*‐MAA)/Ag‐TiO_2_ nanocomposites could be recycled by using a simple procedure. **Figure** **7** illustrates the recycling experiment of the nanocomposites after the adsorption of BF. After the addition of the nanocomposites to the BF solution (Figure 7b), the application of a magnet was sufficient to recover the nanocomposites (Figure 7c), which were able to adsorb most of the BF present in solution.

**Figure 8:**
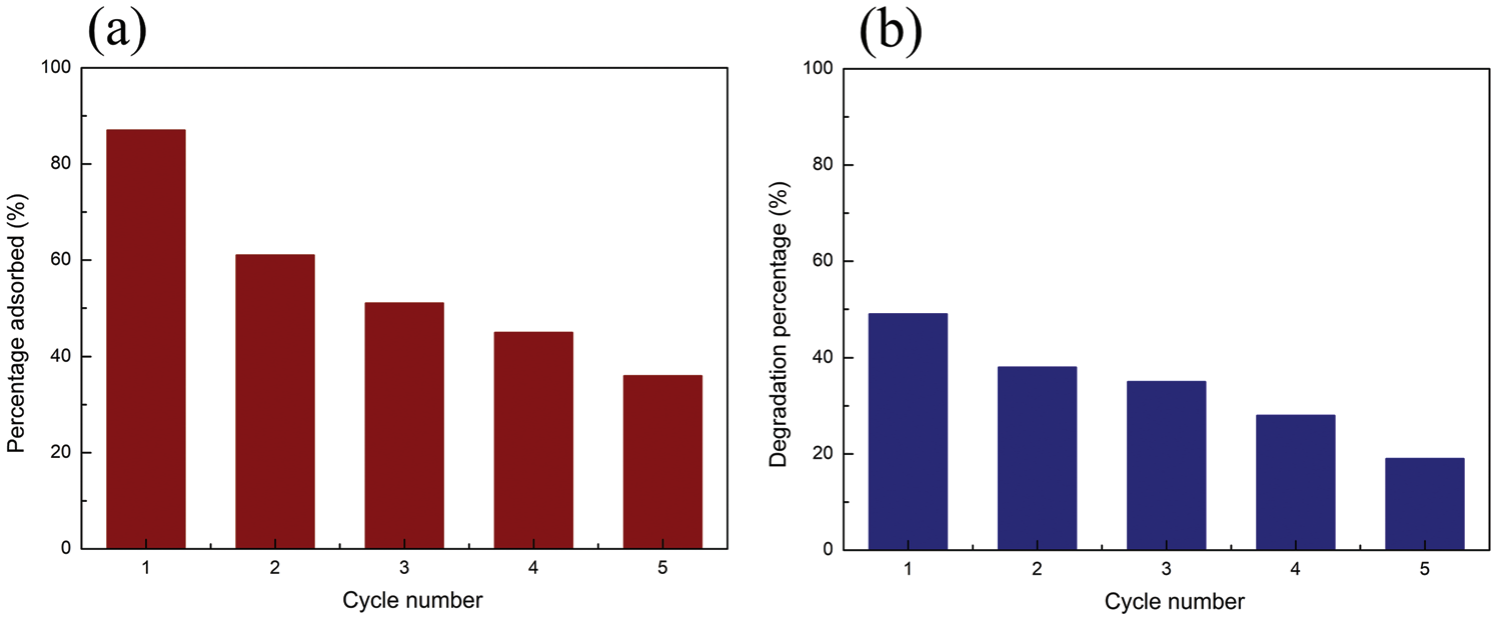
Recycling experiments of Fe_3_O_4_/P(NIPAM‐*co*‐MAA)/Ag‐TiO_2_ nanocomposites a) adsorbing BF under dark conditions and b) degrading CIP under visible‐light irradiation (initial concentration of BF and CIP: 5 mg L^−1^, concentration of the nanocomposites: 460 mg L^−1^).

Besides, the reusability of the nanocomposites in BF adsorption experiments and in CIP visible‐light photocatalytic degradation experiments was investigated. **Figure** **8**a shows that, after five reuse cycles, the adsorption percentage of BF by the nanocomposites decreased from 87% to 36%. Compared to the recycling experiments in the adsorption of BF by a similar system such as the porous Fe_3_O_4_/P(NIPAM‐*co*‐MAA) microspheres^[^ ^27^ ^]^ where the adsorption percentage of BF was about 30% after the fifth cycle, the reusability of the nanocomposites is affected in a similar way showing that the addition of the Ag‐TiO_2_ NPs did not influence the reusability of the system. Figure 8b shows that, after five reuse cycles, the degradation percentage of CIP by the nanocomposites decreased from 49% to 19%. Compared to the five recycling experiments on the degradation of CIP by Ag‐TiO_2_ NPs,^[^ ^28^ ^]^ the reusability of the nanocomposites is decreased. The possible reason is that in the nanocomposites, Ag‐TiO_2_ NPs are linked to the surface of the polymeric microspheres and, therefore, have less available surface area than in the free Ag‐TiO_2_ NPs.

### 2.5 Antibacterial Activity of Fe_3_O_4_/P(NIPAM‐*co*‐MAA)/Ag‐TiO_2_ Nanocomposites

**Figure 9:**
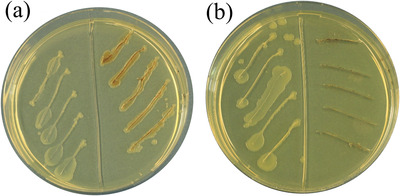
Modified streak method demonstrating the inhibition of *E. coli* growth by Fe_3_O_4_/P(NIPAM‐*co*‐MAA) microspheres and Fe_3_O_4_/P(NIPAM‐*co*‐MAA)/Ag‐TiO_2_ nanocomposites: a) *E. coli* in LB broth on the left part of the plate, microspheres and *E. coli* in LB broth on the right part of the plate; b) *E. coli* in LB broth on the left part of the plate, nanocomposites and *E. coli* in LB broth on the right part of the plate.

The antibacterial properties of Fe_3_O_4_/P(NIPAM‐*co*‐MAA)/Ag‐TiO_2_ nanocomposites toward *E. coli* were qualitatively tested under dark conditions. In **Figure** **9**, the results of a modified streaking method used to test the antibacterial activity of Fe_3_O_4_/P(NIPAM‐*co*‐MAA) microspheres and Fe_3_O_4_/P(NIPAM‐*co*‐MAA)/Ag‐TiO_2_ nanocomposites are shown. *E. coli* in Luria–Bertani (LB) broth were added into every streak on the left part of the plates as a control. Fe_3_O_4_/P(NIPAM‐*co*‐MAA) microspheres and *E. coli* in LB broth were added into every streak on the right part of the plate in Figure 9a. Fe_3_O_4_/P(NIPAM‐*co*‐MAA)/Ag‐TiO_2_ nanocomposites and *E. coli* in LB broth were added into every streak on the right part of the plate in Figure 9b. These two plates were incubated under dark conditions at 37 °C for 24 h. The growth of *E. coli* was similar on the left and right part of the plate in Figure 9a, which indicates that the microspheres cannot inhibit the growth of *E. coli*. Differently, in Figure 9b, there was no growth of *E. coli* on the right part of the plate compared to its left part. These results prove that the nanocomposites can inhibit *E. coli* growth, thanks to the presence of Ag NPs, which are known to have a robust antibacterial activity.^[^ ^28^ ^]^ Furthermore, these results are in line not only with what we previously reported for Ag‐TiO_2_ NPs^[^ ^28^ ^]^ but also with what was reported for other similar systems.^[^ ^43^ ^]^

## 3 Conclusion

In this work, multifunctional Fe_3_O_4_/P(NIPAM‐*co*‐MAA)/Ag‐TiO_2_ nanocomposites were prepared for the first time and characterized. The nanocomposites exhibited an outstanding adsorption capability toward BF, thanks to the presence of the Fe_3_O_4_/P(NIPAM‐*co*‐MAA) microspheres. The adsorption capacity of BF by the nanocomposites could reach 150 mg g^−1^ after 30 min adsorption under dark conditions. The electrostatic interaction between the positively charged BF and the negatively charged carboxylate groups of the nanocomposites plays a crucial role in the BF adsorption process, as it was proved through the study of the adsorption mechanism. In addition, thanks to the presence of Ag‐TiO_2_ NPs, the nanocomposites presented a good visible‐light photocatalytic activity toward CIP and NFX. The degradation rate constant obtained from the pseudo‐first‐order kinetic fitting for CIP and NFX was 3.12 × 10^−3^ min^−1^. Furthermore, the nanocomposites could be easily separated from water, due to their superparamagnetic properties, and reused showing a plausible future industrial application. Finally, the proposed nanocomposites showed, qualitatively, good antibacterial activity toward *E. coli*. Altogether, the nanocomposites presented in this study are promising multifunctional materials to be utilized as adsorbents, photocatalysts, and antibacterial agents, which can find applications in the waste water remediation, especially when complex mixtures of micropollutants are present.

## 4 Experimental Section

### Materials

*N*‐isopropyl acrylamide (NIPAM, 99%), methyl acrylic acid (MAA, 99%), *N,N′*‐methylenebisacrylamide (99%), ammonium persulfate (99%), iron sulfate (FeSO_4_·7H_2_O, 99%), sodium nitrite (NaNO_2_, 97%), ammonia solution (NH_3_·H_2_O, 28 wt% in water), silver nitrate (99%), hydrochloric acid (HCl, 37%), methanol (CH_3_OH, 99.9%), CIP (98%), NFX (98%), BF (80%), and LB broth were purchased from Sigma‐Aldrich (Germany). Titanium dioxide aeroxide TiO_2_ P25 was obtained from Evonik. Agar‐agar was purchased from Merck. The used strain of *E. coli* was as follows: *E. coli* YFP, MG1655 galK::SYFP2‐FRT. Ultrapure water was produced by a MembraPure Astacus system (MembraPure GmbH, Germany).

### Preparation of Fe_3_O_4_/P(NIPAM‐*co*‐MAA)/Ag‐TiO_2_ Nanocomposites

The detailed preparation procedures of Fe_3_O_4_/P(NIPAM‐*co*‐MAA) microspheres were similar to the preparation procedures of Fe_3_O_4_/P(NIPAM‐*co*‐MAA) solid microspheres, which were described in the previously published article.^[^ ^27^ ^]^ The preparation method of Ag‐TiO_2_ NPs was described in a former publication.^[^ ^28^ ^]^ Fe_3_O_4_/P(NIPAM‐*co*‐MAA)/Ag‐TiO_2_ nanocomposites were obtained as follows: 60 mg of Fe_3_O_4_/P(NIPAM‐*co*‐MAA) microspheres were added to 30 mL of ultrapure water and stirred for 30 min at room temperature to ensure the full dispersion of the microspheres in water. Subsequently, 120 mg of Ag‐TiO_2_ NPs were added to the former solution under stirring and room temperature. The mass ratio of Fe_3_O_4_/P(NIPAM‐*co*‐MAA) microspheres and Ag‐TiO_2_ NPs was 1:2. Finally, after 6 h stirring, Fe_3_O_4_/P(NIPAM‐*co*‐MAA)/Ag‐TiO_2_ nanocomposites were obtained by freeze‐drying of the former dispersion.

### Characterization

The morphology and the size of Fe_3_O_4_/P(NIPAM‐*co*‐MAA)/Ag‐TiO_2_ nanocomposites and of Fe_3_O_4_/P(NIPAM‐*co*‐MAA) microspheres were assessed by HR‐TEM. The HR‐TEM images were obtained by using a 200 kV TEM microscope FEI Talos F200X, which was the combination of high‐resolution S/TEM with the EDS signal detection, and 3D chemical characterization with compositional mapping. The microscopic copper grid with a thin transparent carbon film was used as the sample holder for TEM investigations. First, samples with ultrapure water were ultrasonically treated. Immediately after sonification, 3 µL of the solution were dropped on a grid and evaporated at room temperature. Finally, the grid with the deposited samples was stained with phosphotungstic acid and cleaned by UV light irradiation under vacuum before the measurement. The chemical composition of Ag‐TiO_2_ NPs, Fe_3_O_4_/P(NIPAM‐*co*‐MAA) microspheres, and Fe_3_O_4_/P(NIPAM‐*co*‐MAA)/Ag‐TiO_2_ nanocomposites was investigated by ATR‐FTIR spectroscopy. ATR‐FTIR spectra were recorded using an IRAffinity‐1S (Shimadzu, Japan) spectrometer equipped with a GladiATR‐10 accessory (diamond crystal). The spectra were registered in the wavenumber range from 4000 to 500 cm^−1^ with sets of 32 runs and a resolution of 2 cm^−1^. UV–vis spectra of Ag‐TiO_2_ NPs, Fe_3_O_4_/P(NIPAM‐*co*‐MAA) microspheres, and Fe_3_O_4_/P(NIPAM‐*co*‐MAA)/Ag‐TiO_2_ nanocomposites in water were monitored by a Cary 100 Bio UV–vis spectrophotometer (Agilent Technologies, Inc., USA) at room temperature in the wavelength range from 200 to 800 nm. TGA analyses were performed using a thermogravimetric analyzer TGA/DSC 1/1100 SF from Mettler Toledo. 5 mg of Ag‐TiO_2_ NPs, Fe_3_O_4_/P(NIPAM‐*co*‐MAA) microspheres, and Fe_3_O_4_/P(NIPAM‐*co*‐MAA)/Ag‐TiO_2_ nanocomposites were, respectively, heated from 30 to 900 °C (heating rate of 10 °C min^−1^) and under argon atmosphere (with a gas flow rate of 50 mL min^−1^). The magnetic properties of Fe_3_O_4_/P(NIPAM‐*co*‐MAA) microspheres and Fe_3_O_4_/P(NIPAM‐*co*‐MAA)/Ag‐TiO_2_ nanocomposites were investigated by using a VSM (Microsence EV9, USA). The measurements were performed on the powders by applying an external magnetic field from −20 to 20 kOe at room temperature. The final graphs were obtained by dividing the magnetization values for the mass of the samples (about 5.0 mg per sample) used during the experiment.

### Adsorption Experiments

Aqueous solutions with different concentrations of BF (1, 3, 5, 10, 20, 30, 50, 70, 100, 150, 200 mg L^−1^) and at a pH value of 6 were prepared. The pH value of 6 was the natural pH of a solution of the dye BF (at the used concentrations) in ultrapure water without adding an acid or a base. All BF adsorption experiments on the Fe_3_O_4_/P(NIPAM‐*co*‐MAA)/Ag‐TiO_2_ nanocomposites were performed under dark conditions and at room temperature with a constant stirring rate. BF adsorption at different adsorption times was monitored through the BF absorbance changes using a UV–vis spectrophotometer at the BF maximum absorbance (543 nm). For evaluating the adsorption capability of the developed Fe_3_O_4_/P(NIPAM‐*co*‐MAA)/Ag‐TiO_2_ nanocomposites, three main kinds of experiments, namely adsorption kinetic analyses, recycling, and adsorption mechanism experiments were performed. For the adsorption kinetic experiments, 23 mg of the nanocomposites were added to 50 mL of BF solution (BF concentration: 5 mg L^−1^) under stirring in dark conditions. The concentration of the nanocomposites was 460 mg L^−1^. During the adsorption, samples were taken at different adsorption time increments of 5, 10, 15, 20, 30, 40, 50, 60, 70, 80, 90, and 100 min. Before measuring the absorption spectrum of BF at different adsorption times, the samples were centrifuged with an Eppendorf 5417 centrifuge for 20 min at 20 817 × *g* and 20 °C in order to remove the suspended nanocomposites. A similar procedure was followed for the recycling adsorption experiments: after each BF full adsorption experiment, the nanocomposites were recovered by centrifugation (10 min at 20 817 × *g*), washed three times by using 0.1 m HCl, and six times by using ultrapure water to ensure the removal of any adsorbed residue, dried, and reused for the subsequent adsorption experiment of a fresh BF solution. For the investigation on the adsorption mechanism of the nanocomposites toward BF, 23 mg of the nanocomposites were added to each of several BF solutions with different initial concentrations (1, 3, 5, 10, 20, 30, 50, 70, 100, 150, 200 mg L^−1^) under dark conditions and stirring. The volume of BF solution used was 50 mL, and the concentration of the nanocomposites was 460 mg L^−1^. Samples were taken after 100 min adsorption and centrifuged for 20 min at 20 817 × *g* and 20 °C to remove the suspended nanocomposites. The adsorption capacity (*q*_t_, expressed in mg g^−1^) which defined the mass (in mg) of the dye adsorbed by a defined mass of the nanocomposite in g was calculated by using Equation (1):^[^ ^38^, ^42^ ^]^

(1) $$q_{t}=\frac{\left(C_{0}-C_{t}\right)V}{m}$$

where *C*_0_ (mg L^−1^) was the initial concentration of BF, *C_t_* (mg/L) was the concentration of BF after the adsorption time *t* (min), *V* (L) was the volume of BF solution, and *m* (g) was the mass of the nanocomposites.

The pseudo‐first‐order (Equation (2)) and pseudo‐second‐order models (Equation (3)) were used to evaluate the adsorption kinetics of the nanocomposites toward BF.^[^ ^39^ ^]^

(2) $$\ln\left(q_{e}-q_{t}\right)=\ln q_{e}-k_{1}t$$

(3) $$\frac{t}{q_{t}}=\frac{1}{k_{2}q_{e}^{2}}+\frac{t}{q_{e}}$$

In the given formulae, *q*_e_ and *q*_t_ represent the adsorption capacities (mg g^−1^) of the nanocomposites at the adsorption equilibrium and at the time *t* (min), respectively. *k*_1_ was the pseudo‐first‐order rate constant (min^−1^) and *k*_2_ was pseudo‐second‐order rate constant (g mg^−1^ min^−1^). Two well‐known models, that is, the Langmuir isotherm adsorption equation (Equation (4)) and Freundlich (Equation (5)) adsorption models, were used to investigate the adsorption mechanism of the nanocomposites toward BF.^[^ ^27^ ^]^ The selection of these two equations depended on the nature and type of the system, as explained more in detail in the Results and Discussion section.^[^ ^44^, ^45^ ^]^

(4) $$\frac{C_{e}}{q_{e}}=\frac{1}{K_{L}q_{m}}+\frac{C_{e}}{q_{m}}$$

(5) $$\log q_{e}=\log K_{F}+\frac{1}{n}\log C_{e}$$

where *C*_e_ (mg L^−1^) was the equilibrium concentration of the nanocomposites, *q*_e_ (mg g^−1^) was the equilibrium adsorption capacity, *K*_L_ (L mg^−1^) was the Langmuir adsorption equilibrium constant, *q*_m_ (mg g^−1^) was the maximum adsorption capacity, *K*_F_ (mg^1−1/n^ L^1/n^ g^−1^) was the Freundlich adsorption equilibrium constant, and 1/*n* was a constant related to the adsorption intensity.

### Photocatalytic Experiments

As visible‐light source for all the experiments, an artificial lamp produced by Ingenieurbüro Mencke & Tegtmeyer GmbH, Germany, with Susicontrol software (version 2.9.0) and a silicon irradiance sensor to monitor light intensity was used. Its visible light spectrum was similar to the natural solar light with a negligible contribution of UV light (wavelengths range 400–1100 nm, irradiance 98 W m^−2^). The experiments were conducted at a distance between the lamp and the solution surface of 9.4 cm. To evaluate if BF, besides being adsorbed, could also be degraded by photocatalysis under visible‐light irradiation in the presence of the nanocomposites, an additional experiment was performed using only Ag‐TiO_2_ NPs since they were the active photocatalysts in the nanocomposites and since the high adsorption ability of the nanocomposites toward BF complicated the estimation of the photocatalysis contribution to the overall BF removal process. The specific experimental steps were as follows: 15 mg of the Ag‐TiO_2_ NPs were added to 50 mL of the BF solution (5 mg L^−1^) to reach the Ag‐TiO_2_ NPs concentration of 300 mg L^−1^. It was worth noticing that the used concentration of Ag‐TiO_2_ NPs (300 mg L^−1^) corresponded to the concentration of Ag‐TiO_2_ NPs in 460 mg L^−1^ of the nanocomposites. In order to reach the adsorption–desorption equilibrium between the Ag‐TiO_2_ NPs and the BF solution, the solution was kept under stirring in dark conditions for 30 min. Afterward, the solution was irradiated under visible‐light for 180 min. Samples were taken at 0 (before irradiation), 5, 10, 20, 30, 50, 70, 90, 120, 150, and 180 min after visible‐light irradiation. Subsequently, to remove the suspended Ag‐TiO_2_ NPs, these samples were centrifuged for 20 min at 20 817 × *g* and 20 °C. Finally, the absorbance changes of BF at different irradiation times were monitored using a UV–vis spectrophotometer at 543 nm, the maximum absorbance of BF. In order to investigate the photocatalytic activities of Fe_3_O_4_/P(NIPAM‐*co*‐MAA)/Ag‐TiO_2_ nanocomposites toward antibiotics, photodegradation experiments of the antibiotic (either CIP or NFX) were carried out under visible‐light irradiation. The concentration of the antibiotic solution was 5 mg L^−1^ (pH = 3, adjusted with 1 m HCl). The pH of 3 was chosen to ensure better solubility of the antibiotic in water. Before performing the photocatalytic experiments, an adsorption experiment in the dark (dark adsorption) for 180 min with the antibiotics was performed to investigate whether the nanocomposites could also adsorb the antibiotics. The procedure details were as follows: 23 mg of the nanocomposites were added to 50 mL of the antibiotic solution (CIP or NFX, 5 mg L^−1^) under stirring in dark conditions. The concentration of the nanocomposites was 460 mg L^−1^. During the adsorption, samples were taken at adsorption times of 5, 10, 20, 30, 50, 70, 90, 120, 150, and 180 min. Before measuring the absorption spectrum of the antibiotic at different adsorption times, the samples were centrifuged for 20 min at 20 817 × *g* and 20 °C in order to remove the suspended nanocomposites. The photocatalytic experiments were performed as follows: 23 mg of the nanocomposites were added to 50 mL of the antibiotic solution (5 mg L^−1^) to reach the nanocomposites concentration of 460 mg L^−1^. In order to reach the adsorption–desorption equilibrium between the nanocomposites and the antibiotic, the solution was kept under stirring in dark conditions for 30 min. Afterward, the solution was irradiated under visible‐light for 180 min. Samples were taken at 0 (before irradiation), 5, 10, 20, 30, 50, 70, 90, 120, 150, and 180 min after visible‐light irradiation. Subsequently, to remove the suspended nanocomposites, these samples were centrifuged for 20 min at 20 817 × *g* and 20 °C. Finally, the absorbance changes of CIP and NFX at different irradiation times were monitored using a UV–vis spectrophotometer at 277 and 278 nm, the maximum absorbance of CIP and NFX, respectively. A similar procedure was followed for the recycling photocatalytic experiments. After each CIP photodegradation, the nanocomposites were recovered by centrifugation (10 min at 20 817 × *g*). After the centrifugation, the nanocomposites were washed three times with 0.1 m HCl and six times with ultrapure water. Finally, the nanocomposites were dried and reused for the photodegradation of a fresh CIP solution under visible‐light irradiation. The kinetics of the obtained photocatalytic degradation data was analyzed by using a pseudo‐first‐order model (Equation (6)):^[^ ^28^ ^]^

(6) $$-\ln\left(C_{t}/C_{0}\right)=kt$$

where *C*_0_ (mg L^−1^) was the initial concentration of the antibiotic, *C_t_* (mg L^−1^) was the concentration of the antibiotic at the time *t* (min) of irradiation, and *k* (min^−1^) was the pseudo‐first‐order rate constant.

### Antibacterial Activity Experiments

All lab supplies were autoclaved at 121 °C for 20 min, and all experiments were performed under sterile conditions. The procedure for the preparation of *E. coli* suspension culture is described in the following. 10 g of LB broth was mixed with ultrapure water (500 mL) and stirred for 15 min. Next, the LB broth solution was autoclaved. When the LB broth solution was cooled to room temperature, 50 mL of the LB broth solution and 2 mL of *E. coli* culture were mixed in a sterile flask. Finally, this flask was incubated overnight at 37 °C. The preparation of LB agar plate was performed as follows. First, 10 g of LB broth, 7.5 g of agar‐agar, and 500 mL of ultrapure water were mixed and stirred for 15 min. Subsequently, after sterilization and cooling to room temperature, 20 mL of this LB‐agar mixture were added to a sterile Petri dish under a laminar flow chamber. The obtained LB agar plate was stored at 4 °C. The antibacterial activity of the Fe_3_O_4_/P(NIPAM‐*co*‐MAA)/Ag‐TiO_2_ nanocomposites against *E. coli* was tested by using a modified streaking method.^[^ ^28^, ^46^, ^47^ ^]^ First, The LB agar plate was divided into two parts using an antibacterial stick, and five streaks were made for each part. Subsequently, *E. coli* in LB broth solution (20 µL, 10^8^ cells/mL) was transferred to each streak on the left of the plate. Nanocomposites (0.2 mg) together with *E. coli* in LB broth (20 µL, 10^8^ cells/mL) were transferred to each streak on the right part of the plate. The obtained plate was incubated in dark conditions at 37 °C for 24 h to test the growth inhibition of *E. coli* induced by the nanocomposites. For comparison, another identical LB agar plate was divided into two parts, and five streaks were made on each part. The *E. coli* in LB broth solution (20 µL, 10^8^ cells/mL) was transferred to each streak on the left part of the plate. Fe_3_O_4_/P(NIPAM‐*co*‐MAA) microspheres (0.2 mg) together with *E. coli* in LB broth (20 µL, 10^8^ cells/mL) were transferred to each streak on the right part of the plate. The obtained plate was incubated in dark conditions at 37 °C for 24 h to test the growth inhibition of *E. coli* induced by the microspheres.

### Statistical Analysis

The adsorption and photocatalytic data were acquired as two or three replicates and the mean values with corresponding standard deviations were calculated using the Origin software.

## Conflict of Interest

The authors declare no conflict of interest.

## Supporting information

Supporting Information

## Data Availability

The data that support the findings of this study are available from the corresponding author upon reasonable request.
