# Supplementary material for: Reusable and Antibacterial Polymer‐Based Nanocomposites for the Adsorption of Dyes and the Visible‐Light‐Driven Photocatalytic Degradation of Antibiotics
Source: Glob Chall. 2022 Aug 25;6(11):2200076. doi: 10.1002/gch2.202200076 (PMC9638429; doi:10.1002/gch2.202200076)
Supplement: Supplementary file 1 — Supporting Information [file GCH2-6-2200076-s001.pdf]

Global Challenges Open Access

Supporting Information

for *Global Challenges,* DOI: 10.1002/gch2.202200076

Reusable and Antibacterial Polymer-Based Nanocomposites for the Adsorption of Dyes and the Visible-Light-Driven Photocatalytic Degradation of Antibiotics

Jiao Wang, Massimo Sgarzi, Zuzana Němečková, Jiří Henych, Nadia Licciardello, and Gianaurelio Cuniberti**

Supporting Information

Reusable and antibacterial polymer-based nanocomposites for the adsorption of dyes and the visible-light-driven photocatalytic degradation of antibiotics

Jiao Wang, Massimo Sgarzi [∫]*, Zuzana Němečková, Jiří Henych, Nadia Licciardello* [‡]*,* [*] *and Gianaurelio Cuniberti* [*]

J. Wang, M. Sgarzi, N. Licciardello, G. Cuniberti

Institute for Materials Science, Max Bergmann Center of Biomaterials and Dresden Center for Nanoanalysis, TU Dresden, 01062, Dresden, Germany

E-mail of corresponding authors: nadia.licciardello@tu-dresden.de; gianaurelio.cuniberti@tu-dresden.de

Z. Němečková

Institute of Inorganic Chemistry, Czech Academy of Sciences, Husinec-Řež 1001, 250 68 Řež, Czech Republic

J. Henych

Institute of Inorganic Chemistry, Czech Academy of Sciences, Husinec-Řež 1001, 250 68 Řež, Czech Republic and Faculty of Environment, Jan Evangelista Purkyně University, Pasteurova 3632/15, 400 96 Ústí nad Labem, Czech Republic

[∫] Current address of M. Sgarzi: Department of Molecular Sciences and Nanosystems, Ca' Foscari University of Venice, Via Torino 155, 30172 Venezia Mestre, Italy.

[‡]Current address of N. Licciardello: Department of Drug and Health Sciences, University of Catania, Viale Andrea Doria 6, I-95125 Catania, Italy

E-mail: nadia.licciardello@unict.it

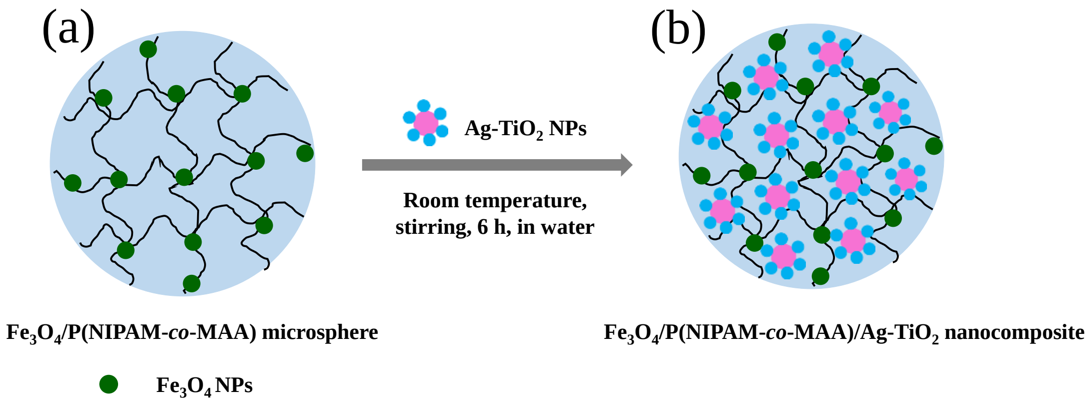

Scheme S1. Preparation process for Fe_3O_4/P(NIPAM-*co*-MAA)/Ag-TiO_2 nanocomposites.

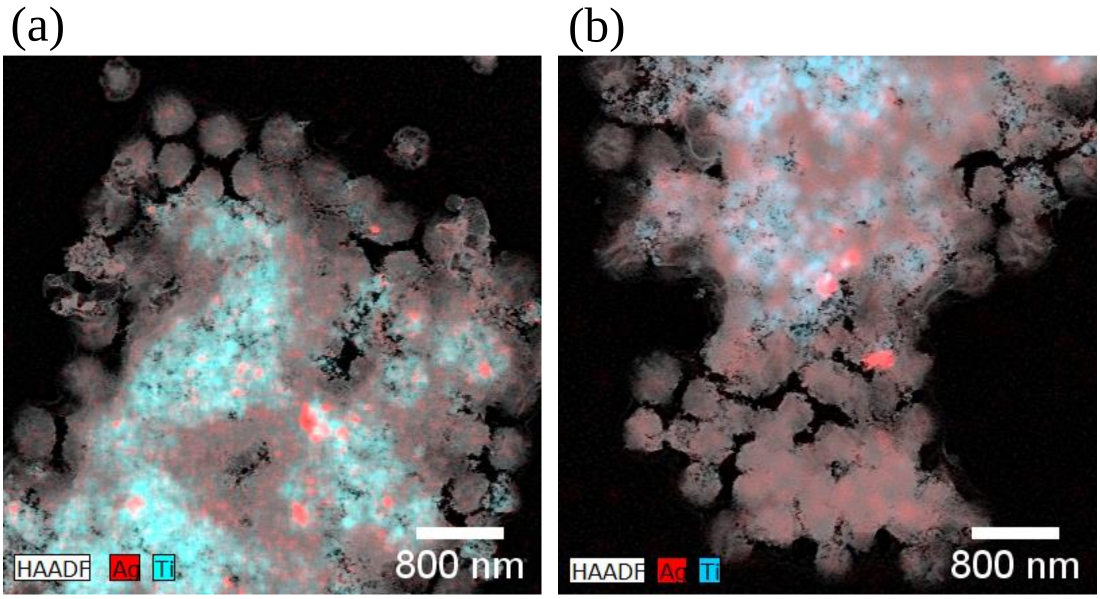

Figure S1. EDS maps of Fe_3O_4/P(NIPAM-*co*-MAA)/Ag-TiO_2 nanocomposites with Ti (blue) and Ag (red) atomic distribution.

Table S1. Quantification of elements performed on Figure S1(a) and (b). The elements W and P come from the staining used during the measurement.

Element	At% (Quantification from Figure S1(a))	At% (Quantification from Figure S1(b))
**Ag**	0.35	0.19
**Ti**	16.96	12.39
**Fe**	3.83	5.05
**C**	18.04	21.55
**O**	47.01	47.40
**W**	12.24	11.77
**P**	1.57	1.66

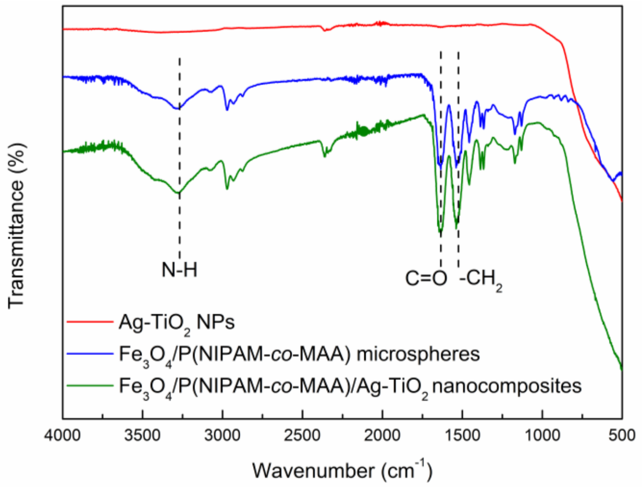

Figure S2. ATR-FTIR spectra of Ag-TiO_2 NPs (red line), Fe_3O_4/P(NIPAM-*co*-MAA) microspheres (blue line) and Fe_3O_4/P(NIPAM-*co*-MAA)/Ag-TiO_2 nanocomposites (green line).

Figure S2 shows the IR spectra of Ag-TiO_2 NPs, Fe_3O_4/P(NIPAM-*co*-MAA) microspheres and Fe_3O_4/P(NIPAM-*co*-MAA)/Ag-TiO_2 nanocomposites. As reported previously,[1] the IR spectrum of Fe_3O_4/P(NIPAM-*co*-MAA) microspheres (blue line) has bands at 1546 and 1650 cm^{-1}, which are ascribable to -CH_2 bending and C=O stretching of MAA, respectively. In addition, the broad band that appears at 3304 cm^{-1}

is characteristic of the N-H stretching of *N*-isopropyl acrylamide and *N*,*N'*-methylenebisacrylamide.

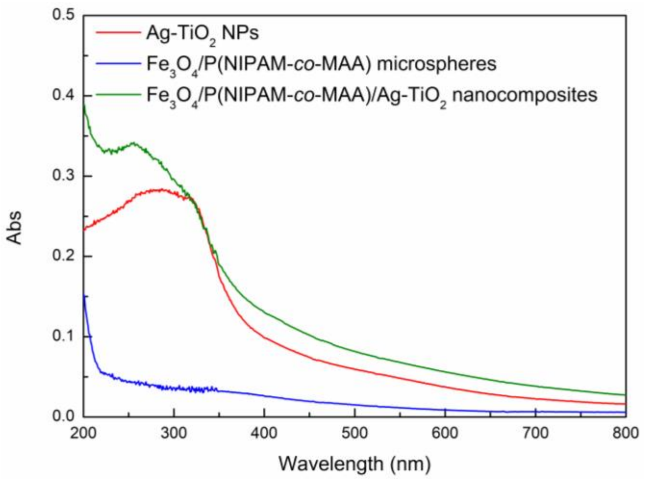

Figure S3. UV-Vis absorption spectra in water of Ag-TiO_2 NPs (red line), Fe_3O_4/P(NIPAM-*co*-MAA) microspheres (blue line) and Fe_3O_4/P(NIPAM-*co*-MAA)/ Ag-TiO_2 nanocomposites (green line).

(a) (b)

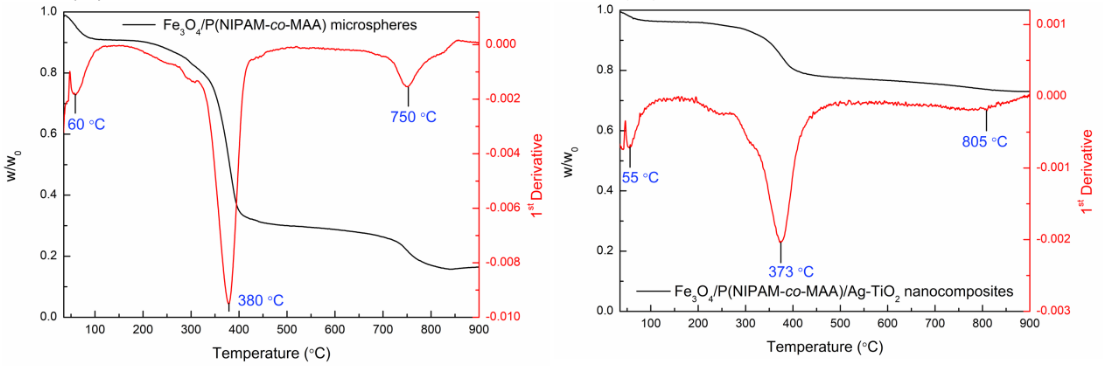

Figure S4. TGA curves with their 1st derivative for (a) Fe_3O_4/P(NIPAM-*co*-MAA) microspheres and (b) Fe_3O_4/P(NIPAM-*co*-MAA)/Ag-TiO_2 nanocomposites.

(a) (b) (c)

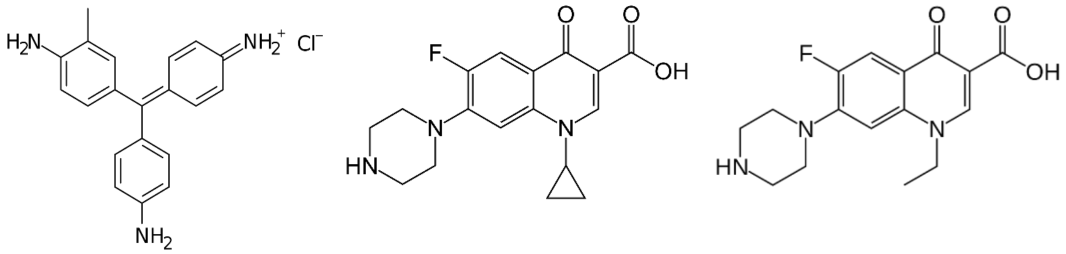

Figure S5. Chemical structure of (a) BF, (b) CIP and (c) NFX.

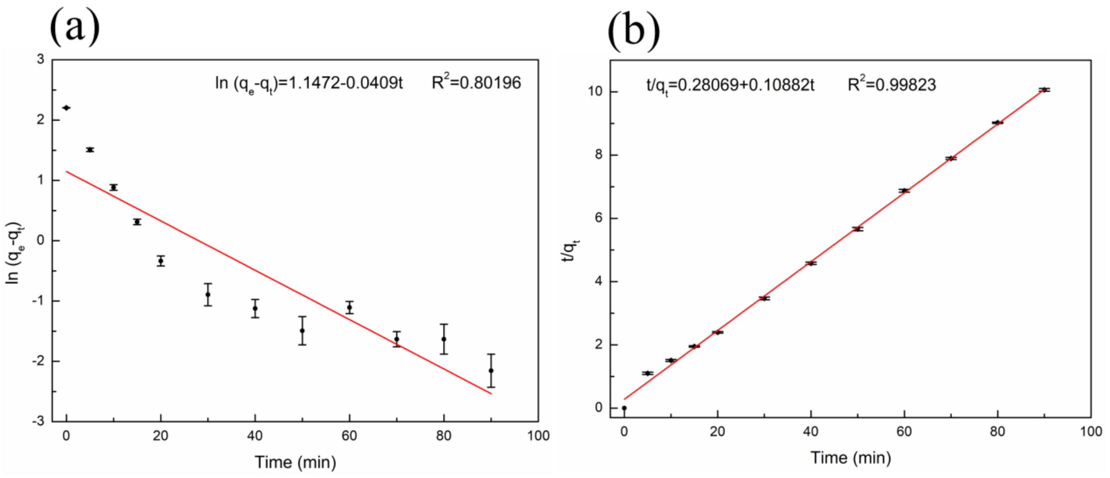

Figure S6. Adsorption kinetics of BF by Fe_3O_4/P(NIPAM-*co*-MAA)/Ag-TiO_2 nanocomposites fitted with (a) pseudo-first-order and (b) pseudo-second-order model (initial concentration of BF: 5 mg/L, concentration of the nanocomposites: 460 mg/L, number of replicates n = 3).

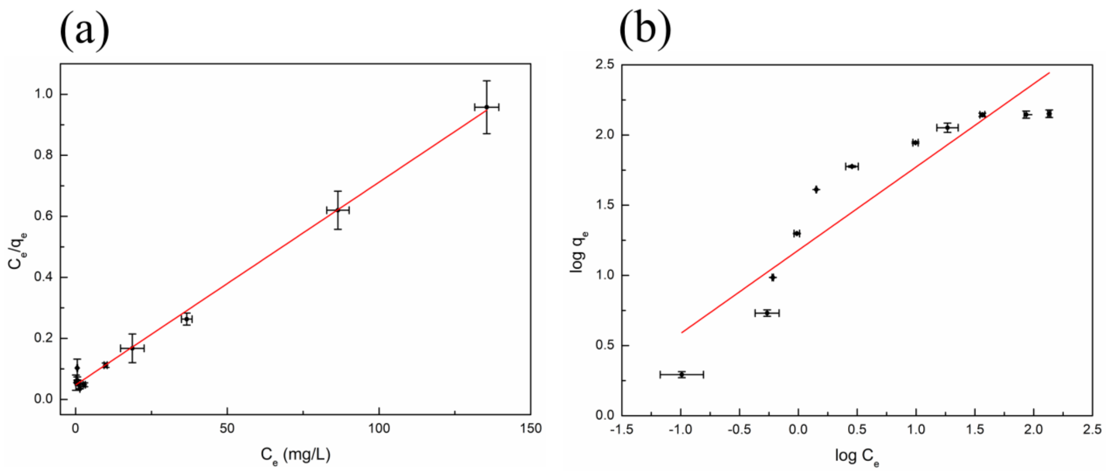

Figure S7. (a) Langmuir model and (b) Freundlich model for the adsorption of BF by Fe_3O_4/P(NIPAM-*co*-MAA)/Ag-TiO_2 nanocomposites (concentration of the nanocomposites: 460 mg/L, number of replicates n = 2).

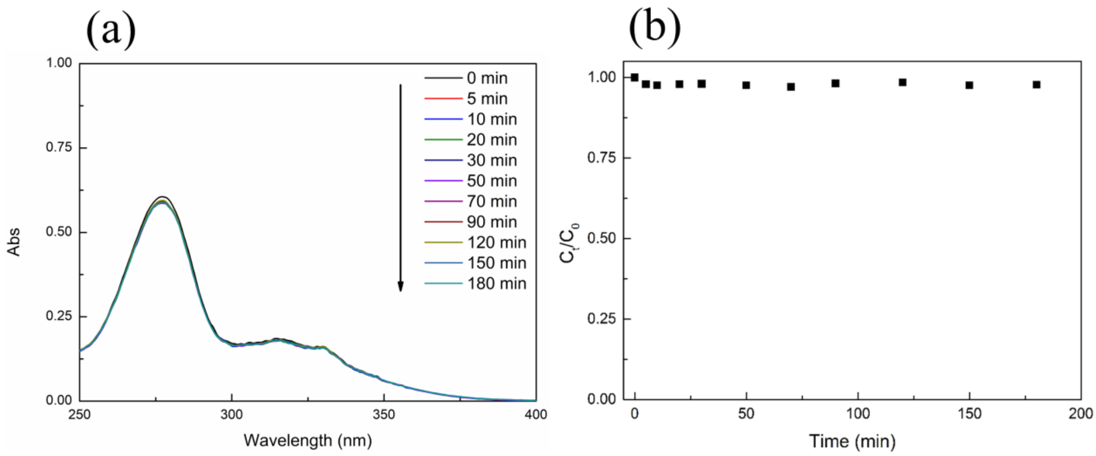

Figure S8. (a) Representative temporal variations of the absorption spectrum of CIP in time during the adsorption on Fe_3O_4/P(NIPAM-*co*-MAA)/Ag-TiO_2 nanocomposites under dark conditions; (b) time-dependent concentration variation of CIP under dark conditions in the presence of Fe_3O_4/P(NIPAM-*co*-MAA)/Ag-TiO_2 nanocomposites (initial concentration of CIP: 5 mg/L, concentration of the nanocomposites: 460 mg/L).

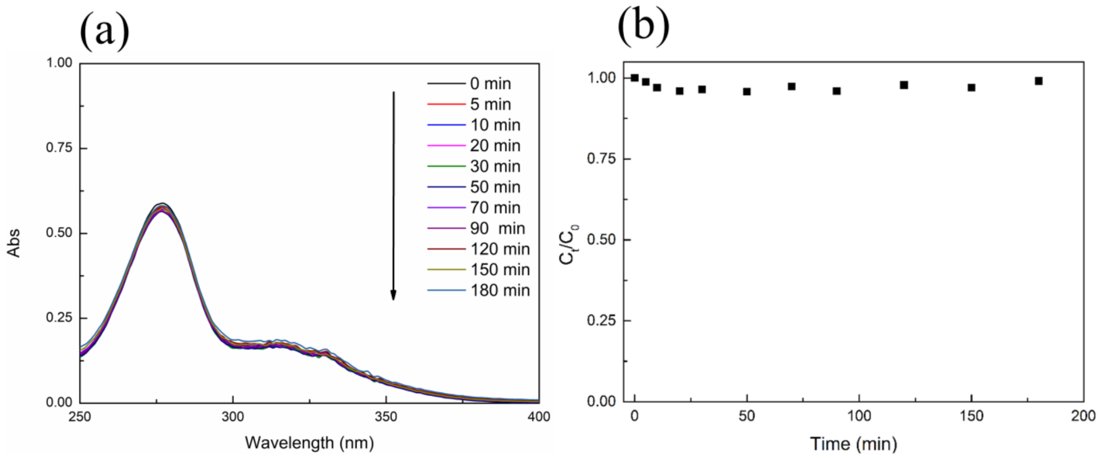

Figure S9. (a) Representative temporal variations of the absorption spectrum of NFX in time during the adsorption on Fe_3O_4/P(NIPAM-*co*-MAA)/Ag-TiO_2 nanocomposites under dark conditions; (b) time-dependent concentration variation of NFX under dark conditions in the presence of Fe_3O_4/P(NIPAM-*co*-MAA)/Ag-TiO_2 nanocomposites (initial concentration of NFX: 5 mg/L, concentration of the nanocomposites: 460 mg/L).

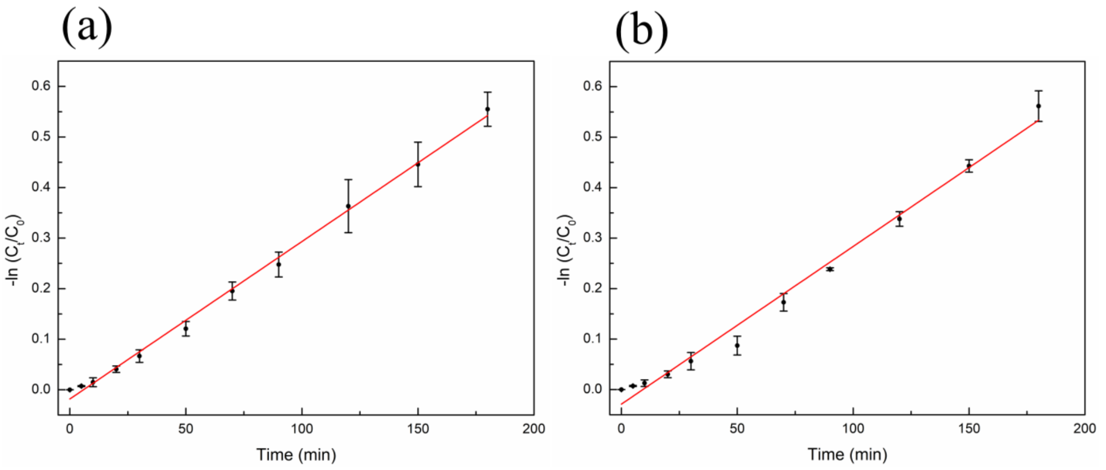

Figure S10. Pseudo-first-order kinetic fitting for (a) CIP solution and (b) NFX solution upon irradiation with visible light in the presence of Fe_3O_4/P(NIPAM-*co*-MAA)/Ag-TiO_2 nanocomposites (initial concentration of CIP or NFX: 5 mg/L, concentration of the nanocomposites: 460 mg/L, number of replicates n = 3).

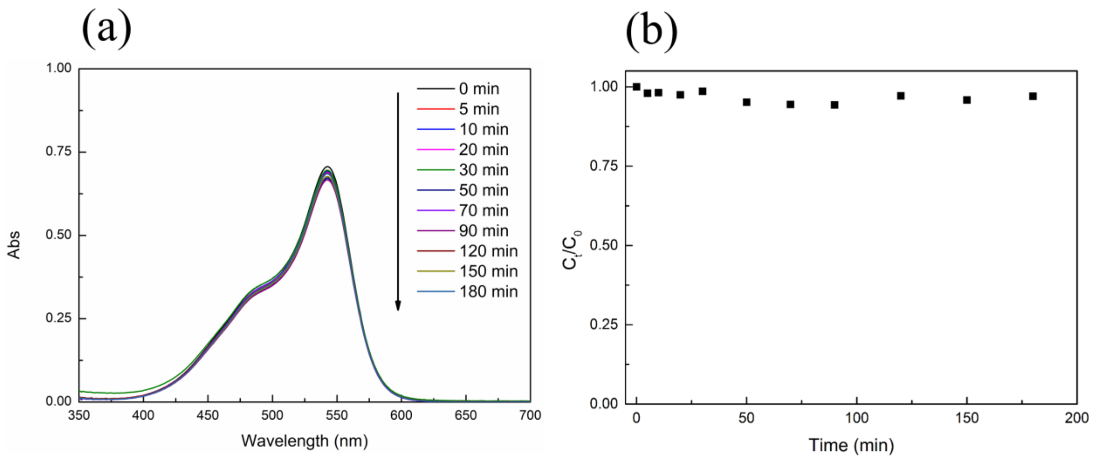

Figure S11. (a) Representative temporal variations of the absorption spectrum of BF under visible-light irradiation in the presence of Ag-TiO_2 NPs; (b) time-dependent variation of the concentration of BF solution upon exposure to visible light in the presence of Ag-TiO_2 NPs (initial concentration of BF: 5 mg/L, concentration of Ag-TiO_2 NPs: 300 mg/L).

References

[1] J. Wang, W. Zhang, Y. Qian, B. Deng, W. Tian, *Macromolecular Materials and Engineering* **2016**, *301*, 1132.
